# Recent Overview of the Use of iPSCs Huntington’s Disease Modeling and Therapy

**DOI:** 10.3390/ijms21062239

**Published:** 2020-03-24

**Authors:** Maria Csobonyeiova, Stefan Polak, Lubos Danisovic

**Affiliations:** 1 Institute of Histology and Embryology, Faculty of Medicine, Comenius University, Sasinkova 4, 811 08 Bratislava, Slovakia; maria.csobonyeiova@fmed.uniba.sk (M.C.); stefan.polak@fmed.uniba.sk (S.P.); 2 Institute of Medical Biology, Genetics and Clinical Genetics, Faculty of Medicine, Comenius University, Sasinkova 4, 811 08 Bratislava, Slovakia; 3 Regenmed Ltd., Medena 29, 811 01 Bratislava, Slovakia

**Keywords:** induced pluripotent stem cells, Huntington’s disease, disease modeling, regeneration

## Abstract

Huntington’s disease (HD) is an inherited, autosomal dominant, degenerative disease characterized by involuntary movements, cognitive decline, and behavioral impairment ending in death. HD is caused by an expansion in the number of CAG repeats in the huntingtin gene on chromosome 4. To date, no effective therapy for preventing the onset or progression of the disease has been found, and many symptoms do not respond to pharmacologic treatment. However, recent results of pre-clinical trials suggest a beneficial effect of stem-cell-based therapy. Induced pluripotent stem cells (iPSCs) represent an unlimited cell source and are the most suitable among the various types of autologous stem cells due to their patient specificity and ability to differentiate into a variety of cell types both in vitro and in vivo. Furthermore, the cultivation of iPSC-derived neural cells offers the possibility of studying the etiopathology of neurodegenerative diseases, such as HD. Moreover, differentiated neural cells can organize into three-dimensional (3D) organoids, mimicking the complex architecture of the brain. In this article, we present a comprehensive review of recent HD models, the methods for differentiating HD–iPSCs into the desired neural cell types, and the progress in gene editing techniques leading toward stem-cell-based therapy.

## 1. Introduction

Huntington’s disease (HD) is one of a group of nine neurodegenerative polyglutamine disorders. It is a devastating and incurable neurological disorder caused by a trinucleotide repeat expansion (CAG) in the gene encoding the huntingtin (HTT) protein, which results in an expanded polyglutamine tract at the N-terminus of the HTT protein. HD is inherited in an autosomal dominant fashion and leads to sleep disturbances, motor dysfunction, cognitive impairment, psychiatric abnormalities, and, ultimately, premature death. Examples of motor dysfunction include involuntary grimacing and excessive gesturing. The neuropsychiatric disorders include disturbances in intellectual and cognitive function, as well as depression, anxiety, aggression, and irritability. The disease affects approximately 1 in 10,000 people. The risk of HD development depends on the number of CAG repeats in the following fashion: less than 35 repeats—minimal risk, 36–40 repeats—moderate risk, and over 40 repeats—high risk. The same direct proportionality also applies to the age of onset (typically 20–65 years) and severity of the disease, which means that a longer CAG repeat sequence is associated with an earlier onset of HD and more severe symptoms [1,2,3]. mHTT impacts multiple physiological processes, such as transcriptional regulation, signal transduction, centrosome formation, and apoptosis. Moreover, mHTT inhibits the transcription and transport of brain-derived neurotrophic factor (BDNF), which is necessary for the survival of striatal neurons, leading to the trophic deprivation of these cells [4]. 

One of the most prominent cytological changes is the formation of insoluble aggregates of the abnormal mHTT protein by its proteolytic cleavage, which is observed as characteristic inclusions within the nucleus and cytoplasm of affected cells. However, the process of toxic mHTT accumulation is not yet clear. Cellular pathologies associated with HD result in the death of striatal GABAergic DARPP-32-positive medium spiny neurons (MSNs) in the basal ganglia, the executive center for organizing motor memory, learning and function in the brain. Striatal atrophy occurs in 95% of HD patients; however, neuronal death is also observed in the cortex, globus pallidus, and thalamic nuclei. Therefore, a promising new therapy is focused on replacing damaged brain tissue using neural stem cells (NSCs) or neural progenitor cells (NPCs) [5]. 

To date, no effective treatment for preventing the onset or progression of HD has been found. Existing therapeutic approaches are only able to alleviate symptoms, and therapies that slow or reverse disease progression have yet to be implemented. Preliminary clinical trials using fetal neural grafts have shown long-lasting functional benefits; however, these grafts were efficient only in limited cases [6,7,8]. 

Stem-cell-based technologies represent a promising direction in HD treatment because of the ability of stem cells to differentiate into NPCs and replace damaged neurons as well as prevent neural cell death. To date, various cell types, such as embryonic stem cells (ESCs), fetal tissue cells, and NSCs have been used in HD cell-based therapy [9]. Despite short-term benefits observed in several patients, graft overgrowth and insufficient survival of the grafted cells hamper the clinical use of this therapeutic approach. Moreover, ethical concerns related to the origin of donor tissue and the limited availability of such cells restrict the use of the above-mentioned cell types. Therefore, induced pluripotent stem cells (iPSCs), which can be derived from any somatic cell type of a patient by using a reprogramming technique, currently represent the most suitable source for personalized cell-based therapy [10,11]. In recent years, a number of research groups have successfully generated patient-specific iPSCs from patients with a neurodegenerative disease, such as Parkinson’s disease, Alzheimer’s disease, amyotrophic lateral sclerosis or Huntington’s disease [12,13]. HD provides an ideal target for iPSC-based gene correction because HD is a monogenic disease with a very well established correlation between the number of CAG repeats and the age of disease onset. This feature allows the production of disease-free cells for potential therapy with autologous cells and provides a valuable platform to study the pathogenesis of the disease [14]. 

Herein, we summarize the recent progress in modeling HD with iPSCs and the possible use of iPSC-differentiated neurons for disease therapy. 

## 2. Differentiation of iPSCs into MSNs

Generally, differentiation protocols are based on recapitulating the developmental steps during ontogenesis. The first step in differentiation is neural induction, including the differentiation of iPSCs into NSCs/NPCs, through embryoid bodies (EBs) and neural rosette formation, which is achieved by inhibiting the bone morphogenic protein (BMP) and transforming growth factor beta (TGF-β) pathways to prevent mesodermal and endodermal differentiation [15]. A comprehensive overview of these protocols can be found in our previous work [16]. 

The second step involves the regional patterning of cells toward a ventral telencephalic fate. Among the inducers utilized, the following morphogens play important roles: sonic hedgehog (SHH), the WNT inhibitor dickkopf 1 (DKK1), and the small-molecule WNT inhibitor XAV939. There are numerous differentiation protocols based on the use of neural differentiation media with the addition of the above-mentioned molecules (Table 1). The first research group that successfully differentiated HD-iPSC-derived NSCs into striatal neurons was Zhang et al. [12], who cultivated HD-iPSC-derived NSCs in medium supplemented with SHH, DKK1, and BDNF and with the addition of the Rho-associated protein kinase (ROCK) inhibitor Y27632 to promote cell survival. The cells were further treated with cAMP and valproic acid. The terminally differentiated HD striatal neurons possessed 72 CAG repeats, the same number as the parental HD fibroblasts. These neurons were positive for elevated caspase activity and expressed the GABAergic neuronal marker GABA. Delli Carri et al. [17] published a protocol for differentiating ESCs/iPSCs into mature MSNs. During the 80-day procedure, through BMP/TGF-β inhibition (involving SHH, DKK1, and BDNF), these researchers were able to induce ventral telencephalic specification of iPSC-derived NPCs, followed by their final maturation into MAP2+/GABA+ neurons. 

Chiu et al. [18] differentiated HD-iPSC NPCs into various types of neurons, including TUJ1-, MAP2-, and Olig2-expressing neurons, by culturing the stem/progenitor cells in N2/B27 media. To enhance the differentiation of the cells toward striatal neurons, the cells were further cultured for 4–6 weeks, resulting in a 70% neural population expressing GABAergic projection neuronal markers. 

Liu et al. [19] generated functional human forebrain GABAergic interneurons from iPSC-derived NPCs in a chemically defined system without requiring transgenic modification or cell sorting. The differentiation of the iPSC-derived NPCs into KX2.1-expressing medial ganglionic eminence progenitors was obtained by simply treating the cells with SHH or its agonist purmorphamine within two weeks. These progenitors were subsequently cultured in neural differentiation medium, giving rise to a population of forebrain GABAergic interneurons by the sixth week. 

The differentiation of GABAergic neurons in a monkey model of HD was published by Carter et al. [20]. In their protocol, mature GABAergic neurons were differentiated from HD–iPSC-derived NPCs by culturing these cells in neural induction medium supplemented with SHH, fibroblast growth factor 8 (FGF8), and ascorbic acid. The successful differentiation was proved by several analyses, which revealed the elevated expression of HTT transcripts, an increased accumulation of mHTT, higher susceptibility to oxidative stress, and the presence of HTT aggregates together with intranuclear inclusions, which are typical characteristics for mature HD neurons. 

Most recently, Cho at al. [21] published a protocol for the differentiation into astrocytes of iPSCs obtained from a non-human primate HD model. The differentiation of the iPSC–NPCs started with their cultivation in medium supplemented with azacytidine (AZA-C), trichostatin (TSA), BMP2, and B27. Later, the AZA-C and TSA were removed, and the differentiation was continued for 28 days in an astrocyte differentiation medium. Over 95% of the mature astrocytes displayed a number of pathologic features associated with HD; therefore, these cells represent an appropriate model of the disease.

## 3. iPSC-Based Modeling of HD

The known genetic mutation in HD allows researchers to create various types of disease models. Widely used animal models ranging from roundworms [24] and Drosophila [25] to large animals, such as sheep, pig [26,27], mouse [28,29], and monkey [30], have been applied to HD modeling. Among these, the most common are murine models, including both knock-in and transgenic models. However, the majority of the mouse models bear a far greater number of CAG repeats (BACHD mouse—97 CAG/CAA; YAC128 mouse—128 CAG; R6/2 mouse—144 CAG repeats) compared with those commonly seen in adult HD patients. Therefore, there is a need for more suitable HD models that avoid the use of animals [31]. 

The iPSC technology not only provides an approach to possible cell-based therapy but also enormously facilitates the investigation of disease pathology and its onset because iPSC-derived NPCs obtained from HD patients harbor the CAG expansion in HTT. To date, a number of studies have reported the usefulness of HD models derived from HD–iPSCs (Table 2). Furthermore, HD–iPSC–NPCs can organize into three-dimensional (3D) organoids mimicking the architecture of brain tissue. In addition, iPSC-derived HD models represent a much more suitable alternative for drug screening and toxicity testing than widely used animal models [15,32]. 

Zhang et al. [12] were among the first authors to generate an iPSC-derived HD model. The researchers developed iPSCs from an HD patient displaying 72 CAG repeats, which were used to generate striatal neurons susceptible to cellular damage with typical characteristics of HD, such as mHTT aggregation and decreased concentrations of glutamate transporters and BDNF. Moreover, the authors tested caspase activity, which is elevated in the post-mortem tissues of HD patients and HD models, to evaluate the ability of HD–iPSC-derived neurons to serve as a model for drug screening. Their results showed an increased caspase activity upon growth factor deprivation, demonstrating the suitability of the HD–iPSC-derived neurons for drug screening. Therefore, these differentiated cells represent a useful human cellular model of HD. An et al. [22] reported the successful targeted correction of an expanded CAG repeat using homologous recombination in iPSCs derived from an HD patient. These corrected HD–iPSCs shared the same genetic background as the diseased cells and thus served as unbiased controls for their uncorrected counterparts. 

Furthermore, the corrected HD–iPSCs normalized the pathogenic signaling pathways and reversed the relevant phenotypic traits, such as the susceptibility to cell death and the altered mitochondrial bioenergetics. The transplantation of the corrected HD–iPSC–NPCs into an HD murine model demonstrated the ability of these cells to survive and differentiate into GABAergic neurons and DARPP-32-positive neurons. Al-Gharaibeh et al. [33] tested the effect of iPSC–NSCs on motor function in the YAC128 mouse model of HD after the bilateral transplantation of these cells into the striata. After 10 weeks, an improved locomotor function was observed according to the results obtained from rotarod testing. Based on the examination of brain tissue specimens, the iPSC–NSCs survived and successfully differentiated into region-specific MSNs within the brain tissue. Moreover, there was no evidence of tumor formation.

Juopperi et al. [2] derived patient-specific iPSC lines from 2 HD patients. The iPSCs from both diseased cell lines were differentiated into glial progenitors and NPCs. Following transplantation of NPCs into the adult mouse brain resulted in their successful engraftment and formation of mature neurons. On the other hand, the HD–iPSC-derived astrocytes exhibited increased cytoplasmic vacuolation in vitro. Similar vacuolations were present within the blood lymphocytes of HD patients; therefore, the authors hypothesized that the vacuolation phenotype is associated with HD. The differentiation of HD–iPSCs into different cellular lineages may reveal novel insights into the cell-specific effects of mHTT. Guo et al. [38] found out that a selective inhibitor (P110-TAT) of the mitochondrial fission protein-dynamin-related protein 1 (DRP1) enhanced mitochondrial function, inhibited an abnormal rate of mitochondrial fragmentation induced by mHTT and increased cell viability in cell culture models of HD. 

Although the HD mutation has been identified, the molecular and cellular basis of HD is less clear. The research group of Szlachcic et al. [34] focused on elucidating the molecular process underlying HD pathology by examining signaling pathways and HD molecular markers in HD–iPSCs from YAC128 mice and humans. Both cell types displayed a number of dysregulated cellular processes, such as ERK signaling, β-catenin phosphorylation, and superoxide dismutase 1 (SOD1) accumulation. Interestingly, the dysregulation of p53 expression was also detected. The presence of these effects in pluripotent cells indicates that the pathological processes begin during early embryonic development. Another group also investigated early molecular pathologies in HD–iPSCs, but with the goal of identifying the pathological, transcriptional changes in juvenile HD–iPSCs, which occur during the neurodevelopment of HD [35]. These authors used the RNA-seq method to study the transcriptional profiles of 6 human juvenile HD–iPSC lines with 71 and 109 CAG repeats. The results revealed numerous significantly dysregulated mRNAs, a large number of which are involved in DNA damage and apoptosis. Based on the prominent upregulation of the vast majority of transcripts, the authors hypothesized that this phenomenon could be related to an overall increase in the expression levels of molecules involved in the pathways essential for the early stages of embryonic development [35]. 

Liu et al. [19] investigated the role of proteasomal activity and the expression of Forkhead box, class (FOXO) transcription factors in the pathology of HD since the FOXO proteins play important roles in longevity, metabolism, cellular proliferation, and stress tolerance. HD–iPSCs and iPSC-derived NPCs were used as disease models. The authors found that HD–iPSCs expressed higher levels of FOXO1 and FOXO4, and FOXO4 overexpression was similarly detected in iPSC-derived NPCs and was associated with elevated proteasomal activity. Their results demonstrated that FOXOs modulate proteasomal activity, and this knowledge can thus be useful in future targeted therapy of HD. 

Another potential target for HD therapy is the A2A adenosine receptor (A2AR) because its stimulation can facilitate neuronal survival [39]. For this reason, Chiu et al. [18] used an HD–iPSC-derived MSN model to study the effect of stimulating A2AR, which is highly distributed in encephalin-expressing MSNs, with selective agonists (CGS21680 and APEC). The HD–iPSC-derived MSNs were treated with both compounds and subsequently exposed to H_2_O_2_. Immunohistochemistry and immunoblotting analyses revealed that CGS21680 and APEC have a neuroprotective effect on HD-iPSC-derived neurons by reducing oxidative stress-induced apoptosis, suggesting the high therapeutic potential of these compounds. 

It is known that calcium homeostasis is closely related to the cellular pathological events in HD. The role of defective calcium signaling during the process of HD progression was investigated by Nekrasov et al. in an iPSC-derived model [23]. The calcium store-operated channel (SOC) currents in iPSC-derived GABA+ MSNs were analyzed via electrophysiological methods. The results revealed that SOC activity in these neurons was increased. This knowledge is useful for investigating potential drugs that can decrease SOC-mediated calcium entry. Therefore, the authors examined the pharmacological effect of the quinazoline derivative EVP4593 on differentiated neurons. The administration of this drug resulted in a positive outcome in terms of reduced activity of SOC currents and the normalization of calcium transport within neurons, thus protecting the neurons from cell death during aging. Based on these findings, the compound EVP4593 represents a possible therapeutic alternative for HD.

During the last decade, researchers have also begun to use monkey models of HD because such models are much more able than murine models to recapitulate the long-term neurodegenerative events in a way that is comparable to that in humans [20,36,37]. 

A monkey model of HD was used by the group of Carter et al. [20] to investigate the effect of pharmacological treatment, specifically memantine (an N-methyl-D-aspartate antagonist), on the HD phenotype. HD GABAergic neurons generated from monkey iPSC-derived NPCs were treated in neural medium supplemented with 10 mM memantine. Cytotoxicity analyses that were performed after 24 h demonstrated the reversal of typical HD pathological events. Similarly, Kunkanjanawan et al. [36] developed iPSC–NPCs from an HD monkey model to examine the therapeutic impact of three drugs, including the above-mentioned memantine and two well-known drugs with a beneficial effect on HD: Rilizole and methylene blue. One day after treatment, the HD–iPSC-derived NPC lines underwent several analyses regarding cytotoxicity, apoptosis, and the formation of mHTT aggregates. According the results obtained, the most potent anti-apoptotic drug was Rilizole, while the most effective drug in the reduction of mHTT aggregates was methylene blue. A positive effect on minimizing cytotoxicity was detected with all three compounds. The results of the above-mentioned studies demonstrate the positive outcome of using non-human primate HD models for analyzing potent therapeutics. 

Recently, Cho et al. [37] published a report on the successful neural differentiation of iPSCs obtained from an HD monkey model (Macaca mulatta) into GABAergic neurons, which were genetically modified by the stable expression of a small-harpin RNA (shRNA) directed against HTT. These neurons were subsequently grafted into the striatum of an HD mouse model. The mouse model lived five weeks longer than did the control group and showed significant improvements in behavioral performance and motor function according to the results obtained from rotarod and grip strength analyses. These optimistic results produce much enthusiasm for the future implementation of gene-correction methods in HD cell-based therapy. 

Another study of Cho et al. [21] deals with the role of astrocytes in the pathogenesis of HD since these glial cells play a significant role in maintaining homeostasis in the central nervous system. The authors generated iPSC–NPCs from a transgenic HD monkey model and differentiated these cells into functional astrocytes that were positivite for astrocyte-specific marker and glial fibrillary acidic protein expression. According to the results of numerous examination techniques, such as fluorescence-activated cell sorting analysis, RT–qPCR, a glutamate uptake assay, and voltage stimulation, the mature astrocytes displayed many cellular phenotypes of HD, including mHTT aggregates, inefficient glutamate clearance, the suppression of mitochondrial function and resistance to oxidative stress and abnormal electrophysiology. Thus, these astrocytes represent a suitable model of HD. Furthermore, the authors investigated the possible therapeutic effect of RNAi on the reduction of HTT expression and the improvement of HD cellular phenotypes. When astrocytes generated from iPSCs obtained from the HD model overexpressed shRNA against HTT, these astrocytes showed a reduction in mHTT aggregates and intracellular inclusions, had restored gene expression and had improved glutamate uptake ability, electrophysiological properties, and cell survival. 

Based on the above-mentioned studies, it is clear that iPSC-based models of HD have enormously improved the methods for studying the pathology that underlies HD on a cellular and molecular level. However, widely used HD iPSC- based models are differentiated in 2D monolayers lacking the tissue- and organ-level structures, which are essential components of the specific tissue microenvironment. Therefore, extensive research has been focused on the formation of more compact 3D cellular models. Comparison of key features between 2D systems and 3D organoids are summarized in table (Table 3).

## 4. iPSC-Derived Brain Organoid Models

The progress in tissue engineering technologies during the last years has eminently improved neurodegenerative disease modeling by the generation of 3D cellular complexes resembling human brain tissue termed brain organoids. Brain organoids derived from pluripotent stem cells can effectively model human cortical development and recapitulate its 3D cytoarchitecture; thus, opening a new window of opportunities to further investigate disease pathogenesis in vitro [40,41]. Moreover, the 3D brain organoids can reveal particular disease phenotypes, which are barely observed in 2D culture systems [42]. For the first time was the concept of whole-brain organoid formation within the same 3D platform published by Lancaster and Knoblich [43]. Within the past years, several brain organoid models of various neural diseases, such as Alzheimer’s disease [44,45] Parkinson’s disease [46,47], neuropsychiatric disease [48], Miller–Dieker syndrome [49], sever microcephaly [50] and also HD [51] have been generated. 

An interesting study published by Conforti et al. [51] provides evidence of the direct link between early neurodevelopmental defects of HD and its adult neurodegenerative phenotype. Using HD iPSC-derived cerebral organoids, the authors repeated stages of ventral telencephalic development and found out that mHTT has a significant negative impact on striatal and cortical specification leading to abnormal neuronal maturation and disrupted cell organization. In the next step, the defect in neuroectodermal development was successfully rescued by allele-specific downregulation of mHTT using a synthetic zinc finger protein (ZFP) repressor.

A similar hypothesis that mHTT may cause early defect during neurodevelopment and neurogenesis led the research team of Zhang et al. [52] to study mention impact of mHTT and CAG repeat length using isogenic HD human ESC/HD iPSC-derived brain organoids. Obtained results from organoids examination showed a lower number of neuroepithelial structures, reduced size, and abnormal development of large radial structures together with the disorganization of neural progenitors. Moreover, the authors demonstrated the negative influence of the protein kinase ataxia telangiectasia mutated (ATM) increased activity on neural cell cycle; therefore, they decided to test the effect of ATM antagonist, KU60019, on neuroepithelium differentiation. The treatment was not fully effective; however, the ATM antagonist significantly improved the proper development of neuroepithelial progenitors. 

Despite the unquestionable advantages of 3D brain organoid models derived from human iPSCs (3D multicellular architecture, organization into cortical layers, long-term culturing, patient-specific disease cell type), there are still several limitations. One of the major challenges is the high structural complexity of the brain tissue. A physiologically relevant brain organoid model needs, for its proper growth and differentiation, a rich vascular network together with immune cells. Several research groups have made efforts trying to address the mentioned issue. For instance, Takabe et al. [53] demonstrated that in vivo transplantation of organ buds generated from neural progenitors and mesenchymal stem cells improved organoid vascularization. Likewise, Mansour et al. [54] detected vascular network formation within human neural organoid after its transplantation into the adult mouse brain. Other differentiation protocols are focused on the development of microglia, the only resident immune cells in CNS [55,56]. Recently, Schwartz et al. [57] established a 3D neural constructs with microglia and vascular network by combining the human pluripotent stem-cell-derived NPCs, endothelial cells, mesenchymal cells, and microglia/macrophage precursors. Such neural constructs highly facilitate neural development within brain organoids. 

Additionally, the technical procedure for the neural organoid formation, conducted by widely used spinning bioreactors, requires constant medium supply over months of culturing, increasing the overall costs [58]. Regarding this issue, a significant improvement was made by the research group of Tachibana et al. [59], who introduced so-called “organoids-on-chip”, which were constructed with the help of biosensors and microfluidic channels, to produce a more suitable, consistent and reproducible culture systems. 

## 5. Gene Therapy for HD

**Figure 1:**
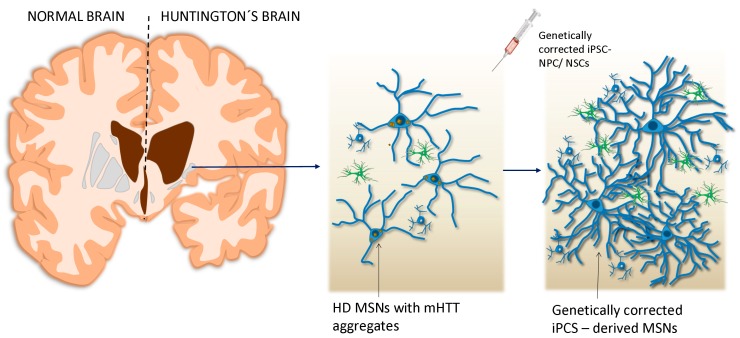
Transplantation of genetically corrected Induced pluripotent stem cells (iPSCs)– neural progenitor cells (NPCs)/neural stem cells (NSCs) into the affected lesion of the brain.

For the possible future use in personalized therapy of iPSC-derived MSNs obtained from an HD patient, the *mHTT* gene must undergo genetic correction (Figure 1). There are several gene silencing/editing approaches, including RNAi, shRNA, antisense oligonucleotides (ASOs), and clustered regularly interspaced short palindromic repeats (CRISPR)/Cas9 [9]. 

However, the major disadvantage of using RNAi and shRNA is the non-specific gene targeting of these approaches, which results in high cell toxicity [60]. In contrast, ASOs specifically suppress the expression of the mHTT allele by targeting a single nucleotide polymorphism (SNP), making this approach more suitable for gene therapy, as demonstrated in murine models [14,61,62]. Nevertheless, partial reduction approaches such as RNAi and ASO rely on SNPs that are specific to the mutant allele, and the normal allele is not readily distinguishable from the expanded allele of the endogenous HTT gene [62,63]. Gene editing approaches such as CRISPR/Cas9 can effectively and permanently eliminate the expression of target genes without the need for continuous administration as required by other techniques. 

An et al. [22] were among the first to publish a report on the successful correction of HD–iPSCs. The CAG repeat in iPSCs was substituted with a normal repeat by homologous recombination resulting in a reduction of polyglutamine repeats to 21. Further differentiation of the corrected cells led to DARPP-32-positive neurons, which exhibited normalized HD signaling pathways and related pathological events. Similar results were obtained by Carroll et al. [64], who developed ASO molecules that were modified with S-constrained-ethyl (cET) motifs to improve selective gene silencing. The potency of these molecules in vivo was examined in adult wild-type mouse models (YAC18 and BACHD). Subsequent Western blot analyses of the striatum revealed a specific knockdown of transgenic human HTT in both model types, suggesting the clinical relevance of the ASO approach.

A novel strategy for the allele-specific genome-editing of mHTT based on the CRISPR/Cas9 technology was published by the groups of Monteys et al. [65] and Shin et al. [66]. Monteys and colleagues developed guide RNAs that bind SpCas9 to six prevalent SNPs located 5′ of HTT exon-1 and successfully eliminated the expression of mHTT. In a similar study, Shin et al. [66] focused on improving allele specificity using allele-specific dual gRNA-mediated CRISPR/Cas9 based on protospacer adjacent motif (PAM)-altering SNPs to specific patient CRISPR/Cas9 sites. The genetic correction was performed on HD–iPSC–NPC lines, which were transfected with 2 µg of CRISPR vectors containing gRNAs. Subsequent analyses revealed a large 44-kb deletion leading to the complete inactivation of mHTT without affecting the normal allele. According to the aforementioned studies and several others [67,68,69], CRISPR/Cas9 has the ability to solely inhibit mHTT expression in a specific brain region and thus has rapidly become the most promising gene-editing tool for neurodegenerative diseases such as HD. However, a question remains regarding the safety of the CRISPR/Cas9 system, which is now being tested in animal models to establish an effective and completely safe procedure before its application in humans [70]. 

## 6. Concluding Remarks

Despite the discovery of a genetic mutation as the main cause of HD, treatment remains mostly aimed at the relief of symptoms and neuroprotection. The lack of clinically-validated targets for this fatal disease places urgency on the need for the development of biologically relevant and clinically predictive models to support the discovery of new targets and drugs. The generation of models based on disease-specific iPSCs tremendously facilitates the progress toward studying HD pathology and the screening of possible therapeutics. However, the complete recapitulation of the neurodegenerative process remains a challenge. The pathologic process of HD develops through several decades of life and does not affect just a single population of cells, but different tissue types. Despite this challenge, researchers are investigating the disease using HD–iPSC-derived MSNs or iPSC-derived astrocytes; however, research that is performed on such a small and specific cell population cannot reflect the complexity of the disease. The solution to this issue could be the generation of 3D brain tissue organoids, which has already been successfully done by several research groups [50,58,71,72]. Currently, the possibility of gene therapy, involving precise gene silencing techniques, has opened a highly promising avenue for personalized cell-based treatment. There are several ongoing trials focused on targeting the abnormal gene and its further effect in eliminating HD. However, an essential condition is to preserve the normal wild-type huntingtin allele, which is critical for maintaining stable neuronal health and neurodevelopment. The recently popularized gene editing method CRISPR/Cas9 possesses this ability, with which it is possible to distinguish the mutant allele from the normal allele and trigger editing of only the mHTT. 

Nevertheless, the safety and efficacy of this approach are in dispute. Finally, yet importantly, it is necessary to note that the reprogramming and differentiation processes also face several challenges. Despite significant progress in reprogramming methods (such as virus-free techniques), the iPSC populations may contain incompletely reprogrammed cells that bear an epigenetic memory of their donor. Analyses of iPSC lines published by Gore et al. [73] revealed a number of acquired non-synonymous, nonsense, and splice variants and epigenetic mutations in genes associated with tumorigenesis. This phenomenon can lead to the insufficient pluripotency of these cells, affecting further neuronal differentiation and possible formation of teratoma and immature astrocytoma from transplanted cells. Therefore, precise purification methods must be established to obtain a completely pure iPSC population. Likewise, the detailed process of the differentiation into the correct cell type of transplanted iPSC–NPCs and their integration into the host microenvironment remains unclear. Therefore, further investigation is required prior to the implementation of preclinical results into clinical practice. Nevertheless, thanks to quite a few pioneering HD studies, we can conclude that HD–iPSCs may represent a key to finding a new treatment option for HD patients. 

## Figures and Tables

**Table 1:** Overview of neural induction and differentiation methods.

Starting Cell Type	Neural Induction	Obtained Cell Type	Final Differentiation	Differentiation Length	Resulting Cell Population	Detected Properties	Reference
HD–iPSCs	Induction of EBs (Neural Expansion Medium + N2/B27 + LIF + bFGF)	NSCs	SHH + DKK1 + BDNF + Y27632 + cAMP and valproic acid	40–42 days	GABA^+^ MSNs	DARPP32 positivity; increased caspase activity	[12]
HD–ES/iPSCs	DMEM/F12 + N2 + SB431542 +Noggin + dorsomorphin	NPCs	SHH + DKK1 + BDNF + N2/B27 + Y27632	80 days	MAP2^+^/GABA^+^ MSNs	DARPP32 positivity; improved behavioral phenotype in lesioned rats	[17]
Human HD–iPSCs	Induction of EBs (DMEM/F1 + N2/B27 + bFGF withdrawal)	NPCs	N2/B27 + NEAA + bFGF	16 weeks	TUJ1^+^, MAP2^+^, and Olig2^+^ neurons; further cultivation into GABA^+^ neurons	GABA/GAD65/DARPP-32 positivity; higher rate of DNA damage	[18]
Human HD–iPSCs	Induction of EBs	NPCs	SHH/purmorphamin + cAMP + BDNF + GDNF and IGF1	60 days	GABA^+^, TUJ1^+^, MSNs	DARPP32 positivity; under exposure to menadion–increased cell death; several small aggregate inclusions	[19]
HD monkey iPSCs	Induction of neural rosettes (DMEM/F12 + N2/B27 + bFGF+ mLIF)	NPCs	SHH/FGF8 and ascorbic acid	43 days	GABA^+^, MAP2^+^ neurons	elevated expression of HTT; presence of HTT aggregates; higher susceptibility to oxidative stress	[20]
HD monkey iPSCs	Neurobasal-A medium + B27+ bFGF + mLIF	NPCs	AZA-C + TSA, BMP2 + B27	30 days	astrocytes	presence of nuclear and cytoplasmic HTT aggregates; higher susceptibility to oxidative stress	[21]
HD–iPSCs	DMEM/F12 + N2 + LIF + bFGF	NPCs	B27 + SHH, DKK1 + BDNF + Y27632	40 days	GABA^+^ neurons	DARPP32 positivity; mHTT genetic correction of pathogenic HD signalling pathways	[22]
HD–iPSCs	DMEM/F12 + N2 + Noggin + Dorsomorphin + bFGF	NPCs	N2/B27 + BDNF + forskolin	56–57 days	GABA^+^ MSNs	Increased protein aggregate inclusions	[23]

**Table 2:** Overview of HD iPSC-derived models.

Model Cell Type	Results	Reference
HD iPSCs–MSNs	- elevated caspase activity upon growth factor deprivation	[12]
HD iPSC–MSN	- neuroprotective effect of CGS21680 and APEC 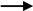 therapeutic potential	[18]
HD iPSC–NPCs	- higher levels of FOXO1 and FOXO4 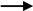 elevated proteasome activity	[19]
iPSC- GABA^+^ neurons	- under treatment with memantine 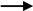 reversal of HD pathologic events	[20]
HD monkey iPSC–astrocytes	- detection of numerous HD related pathologiesm 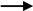 HTT aggregates, inefficient glutamate clearance, suppression of mitochondrial function, abnormal electrophysiology	[21]
Corrected HD iPSC–NPCs	- after transplantation into mice model 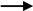 survival and differentiation of cells into the GABAergic neurons	[22]
iPSC–NSCs	- after bilateral transplantation into mice striatum 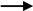 improved locomotor function	[33]
mice HD iPSCs/human HD iPSCs	- dysregulation of ERK signaling, β-catenin phosphorylation, SOD1 accumulation and p53 expression	[34]
Juvenile HD–iPSCs	- high number of significantly dysregulated mRNAs	[35]
HD iPSC–MSN	- increased calcium SOC activity; treatment by quinazoline derivative - EVP4593 led to reduced activity of SOC currents and normalization of calcium transport	[23]
HD monkey iPSC–NPCs	- under treatment with memantine, Rilizole and Methylene blue 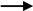 the most potent anti-apoptotic drug was Rilizole; the most effective in reduction of mTT aggregates was Methylene blue	[36]
Corrected HD monkey iPSC–GABA^+^ neurons	- after transplantation into mice striatum 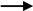 longer lifespan of HD mice model; improved behavioral and locomotor function	[37]

**Table 3:** Comparison between iPSC-derived 2D systems and 3D organoid models.

	2D Systems	3D Organoids
Culture method	- cell growth and differentiation on monolayers	- cell differentiation and self-organization within matrigel
Cell population	- usually immature cell populations	- improved maturation
Duration of differentiation	- fast differentiation process	- slow differentiation process
Tissue composition	- lack of tissue microenvironment	- similar cytoarchitecture with in vivo tissue
Vascular supply	- no	- limited
High-throughput generation	- high	- low
Genome editing	- easy	- hard
Technical procedure	- mostly easy - less time consuming	- moderate - more time consuming
Disease modeling specificity	- moderate	- high

## References

[1] Kaye J.A., Finkbeiner S. (2013). Modeling Huntington’s disease with induced pluripotent stem cells. Mol. Cell. Neurosci..

[2] Juopperi T.A., Kim W.R., Chiang C.H., Yu H., Margolis R.L., Ross C.A., Ming G.L., Song H. (2012). Astrocytes generated from patient induced pluripotent stem cells recapitulate features of Huntington’s disease patient cells. Mol. Brain.

[3] Wyant K.J., Ridder A.J., Dayalu P. (2017). Huntington’s Disease-Update on Treatments. Curr. Neurol. Neurosci. Rep..

[4] Bates G.P., Dorsey R., Gusella J.F., Hayden M.R., Kay C., Leavitt B.R., Nance M., Ross C.A., Scahill R.I., Wetzel R. (2015). Huntington’s disease. Nat. Rev. Dis. Primers.

[5] Smith D.K., He M., Zhang C.L., Zheng J.C. (2017). The therapeutic potential of cell identity reprogramming for the treatment of aging-related neurodegenerative disorders. Prog. Neurobiol..

[6] Bachoud-Lévi A.C., Gaura V., Brugières P., Lefaucheur J.P., Boissé M.F., Maison P., Baudic S., Ribeiro M.J., Bourdet C., Remy P. (2006). Effect of fetal neural transplants in patients with Huntington’s disease 6 years after surgery: A long-term follow-up study. Lancet Neurol..

[7] Carter R.L., Chan A.W. (2012). Pluripotent stem cells models for Huntington’s disease: Prospects and challenges. J. Genet. Genom..

[8] Haddad M.S., Wenceslau C.V., Pompeia C., Kerkis I. (2016). Cell-based technologies for Huntington’s disease. Dement. Neuropsychol..

[9] Chen Y., Carter R.L., Cho I.K., Chan A.W. (2014). Cell-based therapies for Huntington’s disease. Drug Discov. Today.

[10] Bachoud-Lévi A.C. (2017). From open to large-scale randomized cell transplantation trials in Huntington’s disease: Lessons from the multicentric intracerebral grafting in Huntington’s disease trial (MIG-HD) and previous pilot studies. Prog. Brain Res..

[11] Golas M.M. (2018). Human cellular models of medium spiny neuron development and Huntington disease. Life Sci..

[12] Zhang N., An M.C., Montoro D., Ellerby L.M. (2010). Characterization of Human Huntington’s Disease Cell Model from Induced Pluripotent Stem Cells. PLoS Curr..

[13] Camnasio S., Delli Carri A., Lombardo A., Grad I., Mariotti C., Castucci A., Rozell B., Lo Riso P., Castiglioni V., Zuccato C. (2012). The first reported generation of several induced pluripotent stem cell lines from homozygous and heterozygous Huntington’s disease patients demonstrates mutation related enhanced lysosomal activity. Neurobiol. Dis..

[14] Zhang Y., Friedlander R.M. (2011). Using non-coding small RNAs to develop therapies for Huntington’s disease. Gene Ther..

[15] Geater C., Hernandez S., Thompson L., Mattis V.B. (2018). Cellular Models: HD Patient-Derived Pluripotent Stem Cells. Methods Mol. Biol..

[16] Csobonyeiova M., Polak S., Zamborsky R., Danisovic L. (2019). Recent Progress in the Regeneration of Spinal Cord Injuries by Induced Pluripotent Stem Cells. Int. J. Mol. Sci..

[17] Delli Carri A., Onorati M., Castiglioni V., Faedo A., Camnasio S., Toselli M., Biella. G., Cattaneo E. (2013). Human pluripotent stem cell differentiation into authentic striatal projection neurons. Stem Cell Rev. Rep..

[18] Chiu F.L., Lin J.T., Chuang C.Y., Chien T., Chen C.M., Chen K.H., Hsiao H.Y., Lin Y.S., Chern Y., Kuo H.C. (2015). Elucidating the role of the A2A adenosine receptor in neurodegeneration using neurons derived from Huntington’s disease iPSCs. Hum. Mol. Genet..

[19] Liu Y., Qiao F., Leiferman P.C., Ross A., Schlenker E.H., Wang H. (2017). FOXOs modulate proteasome activity in human-induced pluripotent stem cells of Huntington’s disease and their derived neural cells. Hum. Mol. Genet..

[20] Carter R.L., Chen Y., Kunkanjanawan T., Xu Y., Moran S.P., Putkhao K., Yang J., Huang A.H., Parnpai R., Chan A.W. (2014). Reversal of cellular phenotypes in neural cells derived from Huntington’s disease monkey-induced pluripotent stem cells. Stem Cell Rep..

[21] Cho I.K., Yang B., Forest C., Qian L., Chan A.W.S. (2019). Amelioration of Huntington’s disease phenotype in astrocytes derived from iPSC-derived neural progenitor cells of Huntington’s disease monkeys. PLoS ONE.

[22] An M.C., Zhang N., Scott G., Montoro D., Wittkop T., Mooney S., Melov S., Ellerby L.M. (2012). Genetic correction of Huntington’s disease phenotypes in induced pluripotent stem cells. Cell Stem Cell.

[23] Nekrasov E.D., Vigont V.A., Klyushnikov S.A., Lebedeva O.S., Vassina E.M., Bogomazova A.N., Chestkov I.V., Semashko T.A., Kiseleva E., Suldina L.A. (2016). Manifestation of Huntington’s disease pathology in human induced pluripotent stem cell-derived neurons. Mol. Neurodegener..

[24] Li J., Le W. (2013). Modeling neurodegenerative diseases in Caenorhabditis elegans. Exp. Neurol..

[25] Green E.W., Giorgini F. (2012). Choosing and using Drosophila models to characterize modifiers of Huntington’s disease. Biochem. Soc. Trans..

[26] Yang D., Wang C.E., Zhao B., Li W., Ouyang Z., Liu Z., Yang H., Fan P., O’Neill A., Gu W. (2010). Expression of Huntington’s disease protein results in apoptotic neurons in the brains of cloned transgenic pigs. Hum. Mol. Genet..

[27] Ardan T., Baxa M., Levinská B., Sedláčková M., Nguyen T.D., Klíma J., Juhás Š., Juhásová J., Šmatlíková P., Vochozková P. (2019). Transgenic minipig model of Huntington’s disease exhibiting gradually progressing neurodegeneration. Dis. Models Mech..

[28] Figiel M., Szlachcic W.J., Switonski P.M., Gabka A., Krzyzosiak W.J. (2012). Mouse models of polyglutamine diseases: Review and data table. Part I. Mol. Neurobiol..

[29] Switonski P.M., Szlachcic W.J., Gabka A., Krzyzosiak W.J., Figiel M. (2012). Mouse models of polyglutamine diseases in therapeutic approaches: Review and data table. Part II. Mol. Neurobiol..

[30] Yang S.H., Cheng P.H., Banta H., Piotrowska-Nitsche K., Yang J.J., Cheng E.C., Snyder B., Larkin K., Liu J., Orkin J. (2008). Towards a transgenic model of Huntington’s disease in a non-human primate. Nature.

[31] Chang R., Liu X., Li S., Li X.J. (2015). Transgenic animal models for study of the pathogenesis of Huntington’s disease and therapy. Drug Des. Dev. Ther..

[32] Bordoni M., Rey F., Fantini V., Pansarasa O., Di Giulio A.M., Carelli S., Cereda C. (2018). From Neuronal Differentiation of iPSCs to 3D Neuro-Organoids: Modelling and Therapy of Neurodegenerative Diseases. Int. J. Mol. Sci..

[33] Al-Gharaibeh A., Culver R., Stewart A.N., Srinageshwar B., Spelde K., Frollo L., Kolli N., Story D., Paladugu L., Anwar S. (2017). Induced Pluripotent Stem Cell-Derived Neural Stem Cell Transplantations Reduced Behavioral Deficits and Ameliorated Neuropathological Changes in YAC128 Mouse Model of Huntington’s Disease. Front. Neurosci..

[34] Szlachcic W.J., Switonski P.M., Krzyzosiak W.J., Figlerowicz M., Figiel M. (2015). Huntington disease iPSCs show early molecular changes in intracellular signaling, the expression of oxidative stress proteins and the p53 pathway. Dis. Models Mech..

[35] Świtońska K., Szlachcic W.J., Handschuh L., Wojciechowski P., Marczak Ł., Stelmaszczuk M., Figlerowicz M., Figiel M. (2019). Identification of Altered Developmental Pathways in Human Juvenile HD iPSC With 71Q and 109Q Using Transcriptome Profiling. Front. Cell. Neurosci..

[36] Kunkanjanawan T., Carter R., Ahn K.S., Yang J., Parnpai R., Chan A.W.S. (2017). Induced Pluripotent HD Monkey Stem Cells Derived Neural Cells for Drug Discovery. SLAS Discov..

[37] Cho I.K., Hunter C.E., Ye S., Pongos A.L., Chan A.W.S. (2019). Combination of stem cell and gene therapy ameliorates symptoms in Huntington’s disease mice. NPJ Regen. Med..

[38] Guo X., Disatnik M.H., Monbureau M., Shamloo M., Mochly-Rosen D., Qi X. (2013). Inhibition of mitochondrial fragmentation diminishes Huntington’s disease-associated neurodegeneration. J. Clin. Investig..

[39] Popoli P., Blum D., Domenici M.R., Burnouf S., Chern Y. (2008). A critical evaluation of adenosine A2A receptors as potentially “druggable” targets in Huntington’s disease. Curr. Pharm. Des..

[40] Pacitti D., Privolizzi R., Bax B.E. (2019). Organs to Cells and Cells to Organoids: The Evolution of in vitro Central Nervous System Modelling. Front. Cell. Neurosci..

[41] Wu Y.Y., Chiu F.L., Yeh C.S., Kuo H.C. (2019). Opportunities and challenges for the use of induced pluripotent stem cells in modelling neurodegenerative disease. R. Soc. Open Biol..

[42] Zhang X., Hu D., Shang Y., Qi X. (2020). Using induced pluripotent stem cell neuronal models to study neurodegenerative diseases. Biochim. Biophys. Acta Mol. Basis Dis..

[43] Lancaster M.A., Knoblich J.A. (2014). Generation of cerebral organoids from human pluripotent stem cells. Nat. Protoc..

[44] Zhang D., Pekkanen-Mattila M., Shahsavani M., Falk A., Teixeira A.I., Herland A. (2014). A 3D Alzheimer’s disease culture model and the induction of P21-activated kinase mediated sensing in iPSC derived neurons. Biomaterials.

[45] Raja W.K., Mungenast A.E., Lin Y.T., Ko T., Abdurrob F., Seo J., Tsai L.H. (2016). Self-Organizing 3D Human Neural Tissue Derived from Induced Pluripotent Stem Cells Recapitulate Alzheimer’s Disease Phenotypes. PLoS ONE.

[46] Son M.Y., Sim H., Son Y.S., Jung K.B., Lee M.O., Oh J.H., Chung S.K., Jung C.R., Kim J. (2017). Distinctive genomic signature of neural and intestinal organoids from familial Parkinson’s disease patient-derived induced pluripotent stem cells. Neuropathol. Appl. Neurobiol..

[47] Kim H., Park H.J., Choi H., Chang Y., Park H., Shin J., Kim J., Lengner C.J., Lee Y.K., Kim J. (2019). Modeling G2019S-LRRK2 Sporadic Parkinson’s Disease in 3D Midbrain Organoids. Stem Cell Rep..

[48] Srikanth P., Lagomarsino V.N., Muratore C.R., Ryu S.C., He A., Taylor W.M., Zhou C., Arellano M., Young-Pearse T.L. (2018). Shared effects of DISC1 disruption and elevated WNT signaling in human cerebral organoids. Transl. Psychiatry.

[49] Iefremova V., Manikakis G., Krefft O., Jabali A., Weynans K., Wilkens R., Marsoner F., Brändl B., Müller F.J., Koch P. (2017). An Organoid-Based Model of Cortical Development Identifies Non-Cell-Autonomous Defects in Wnt Signaling Contributing to Miller-Dieker Syndrome. Cell Rep..

[50] Lancaster M.A., Renner M., Martin C.A., Wenzel D., Bicknell L.S., Hurles M.E., Homfray T., Penninger J.M., Jackson A.P., Knoblich J.A. (2013). Cerebral organoids model human brain development and microcephaly. Nature.

[51] Conforti P., Besusso D., Bocchi V.D., Faedo A., Cesana E., Rossetti G., Ranzani V., Svendsen C.N., Thompson L.M., Toselli M. (2018). Faulty neuronal determination and cell polarization are reverted by modulating HD early phenotypes. Proc. Natl. Acad. Sci. USA.

[52] Zhang J., Ooi J., Utami K.H., Langley S.R., Aning O.A., Park D.S., Renner M., Ma S., Cheok C.F., Knoblich J.A. (2020). Expanded huntingtin CAG repeats disrupt the balance between neural progenitor expansion and differentiation in human cerebral organoids. bioRxiv.

[53] Takebe T., Enomura M., Yoshizawa E., Kimura M., Koike H., Ueno Y., Matsuzaki T., Yamazaki T., Toyohara T., Osafune K. (2015). Vascularized and Complex Organ Buds from Diverse Tissues via Mesenchymal Cell-Driven Condensation. Cell Stem Cell.

[54] Mansour A.A., Gonçalves J.T., Bloyd C.W., Li H., Fernandes S., Quang D., Johnston S., Parylak S.L., Jin X., Gage F.H. (2018). An in vivo model of functional and vascularized human brain organoids. Nat. Biotechnol..

[55] Ormel P.R., de Sá Vieira R., van Bodegraven E.J., Karst H., Harschnitz O., Sneeboer M.A.M., Johansen L.E., van Dijk R.E., Scheefhals N., Berdenis van Berlekom A. (2018). Microglia innately develop within cerebral organoids. Nat. Commun..

[56] Pandya H., Shen M.J., Ichikawa D.M., Sedlock A.B., Choi Y., Johnson K.R., Kim G., Brown M.A., Elkahloun A.G., Maric D. (2017). Differentiation of human and murine induced pluripotent stem cells to microglia-like cells. Nat. Neurosci..

[57] Schwartz M.P., Hou Z., Propson N.E., Zhang J., Engstrom C.J., Santos Costa V., Jiang P., Nguyen B.K., Bolin J.M., Daly W. (2015). Human pluripotent stem cell-derived neural constructs for predicting neural toxicity. Proc. Natl. Acad. Sci. USA.

[58] Costamagna G., Andreoli L., Corti S., Faravelli I. (2019). iPSCs-Based Neural 3D Systems: A Multidimensional Approach for Disease Modeling and Drug Discovery. Cells.

[59] Tachibana C.Y. (2018). Stem-cell culture moves to the third dimension. Nature.

[60] McBride J.L., Boudreau R.L., Harper S.Q., Staber P.D., Monteys A.M., Martins I., Gilmore B.L., Burstein H., Peluso R.W., Polisky B. (2008). Artificial miRNAs mitigate shRNA-mediated toxicity in the brain: Implications for the therapeutic development of RNAi. Proc. Natl. Acad. Sci. USA.

[61] Drouet V., Perrin V., Hassig R., Dufour N., Auregan G., Alves S., Bonvento G., Brouillet E., Luthi-Carter R., Hantraye P. (2009). Sustained effects of nonallele-specific Huntingtin silencing. Ann. Neurol..

[62] Kordasiewicz H.B., Stanek L.M., Wancewicz E.V., Mazur C., McAlonis M.M., Pytel K.A., Artates J.W., Weiss A., Cheng S.H., Shihabuddin L.S. (2012). Sustained therapeutic reversal of Huntington’s disease by transient repression of huntingtin synthesis. Neuron.

[63] Sah D.W., Aronin N. (2011). Oligonucleotide therapeutic approaches for Huntington disease. J. Clin. Investig..

[64] Carroll J.B., Warby S.C., Southwell A.L., Doty C.N., Greenlee S., Skotte N., Hung G., Bennett C.F., Freier S.M., Hayden M.R. (2011). Potent and selective antisense oligonucleotides targeting single-nucleotide polymorphisms in the Huntington disease gene/allele-specific silencing of mutant huntingtin. Mol. Ther..

[65] Monteys A.M., Ebanks S.A., Keiser M.S., Davidson B.L. (2017). CRISPR/Cas9 Editing of the Mutant Huntingtin Allele In Vitro and In Vivo. Mol. Ther..

[66] Shin J.W., Kim K.H., Chao M.J., Atwal R.S., Gillis T., MacDonald M.E., Gusella J.F., Lee J.M. (2016). Permanent inactivation of Huntington’s disease mutation by personalized allele-specific CRISPR/Cas9. Hum. Mol. Genet..

[67] Yang S., Chang R., Yang H., Zhao T., Hong Y., Kong H.E., Sun X., Qin Z., Jin P., Li S. (2017). CRISPR/Cas9-mediated gene editing ameliorates neurotoxicity in mouse model of Huntington’s disease. J. Clin. Investig..

[68] Kolli N., Lu M., Maiti P., Rossignol J., Dunbar G.L. (2017). CRISPR-Cas9 Mediated Gene-Silencing of the Mutant Huntingtin Gene in an In Vitro Model of Huntington’s Disease. Int. J. Mol. Sci..

[69] Tabrizi S.J., Ghosh R., Leavitt B.R. (2019). Huntingtin Lowering Strategies for Disease Modification in Huntington’s Disease. Neuron.

[70] Kay C., Skotte N.H., Southwell A.L., Hayden M.R. (2014). Personalized gene silencing therapeutics for Huntington disease. Clin. Genet..

[71] Di Lullo E., Kriegstein A.R. (2017). The use of brain organoids to investigate neural development and disease. Nat. Rev. Neurosci..

[72] Bagley J.A., Reumann D., Bian S., Lévi-Strauss J., Knoblich J.A. (2017). Fused cerebral organoids model interactions between brain regions. Nat. Methods.

[73] Gore A., Li Z., Fung H.L., Young J.E., Agarwal S., Antosiewicz-Bourget J., Canto I., Giorgetti A., Israel M.A., Kiskinis E. (2011). Somatic coding mutations in human induced pluripotent stem cells. Nature.

